# Food Price Changes, Household Food and Nutrition Security in Ethiopia: Evidence From Household Level Analysis Through a Gender Lens

**DOI:** 10.1002/fsn3.70685

**Published:** 2025-07-23

**Authors:** Abule Mehare, Lamessa T. Abdisa, Shemelis Kebede Hundie

**Affiliations:** ^1^ Ethiopian Economics Association Addis Ababa Ethiopia; ^2^ Doctoral School of Regional Policy and Economics, Faculty of Business and Economics University of Pécs Pécs Hungary

**Keywords:** Ethiopia, food price, food security, gender inequality, nutrition, random effect

## Abstract

This study investigates the impact of food price changes on food security and diet quality in Ethiopia using recent Living Standard Measurement Surveys. Household‐level food security and diet quality were assessed using the Household Diet Diversity Score, Food Insecurity Experience Scale, and proportion of food consumption expenditure. Employing a random effects panel regression and Oaxaca–Blinder decomposition, this study examined the differential effects of price changes across household types, guided by the Harvard gender analysis framework. The results of the study show that food prices significantly influence household food expenditure shares. Rising prices force households to allocate a greater income portion to food, often reducing non‐food expenditures, especially for vulnerable groups such as female‐headed and low‐income households. Urban households with better access to diverse food and higher incomes exhibit lower foods expenditure shares than rural households. Dietary diversity declined, particularly among female‐headed households and those aged 26–49, highlighting the influence of household size, income, and resource access. The findings of this study underscore the critical importance of addressing food price rises and their consequences for household food security in Ethiopia. Policy interventions aimed at stabilizing food prices, promoting sustainable agricultural practices, and enhancing social safety nets are crucial for mitigating the adverse impacts of food price shocks on vulnerable populations. Furthermore, targeted interventions aimed at empowering women and addressing gender inequalities in access to resources and decision‐making can help improve food security and nutrition outcomes for female‐headed households.

## Graphical abstract

The study reveals that rising food prices disproportionately impact vulnerable groups, notably female‐headed and low‐income households, by increasing food expenditure shares and reducing dietary diversity. Households aged 26–49 experience notable declines in diet quality, influenced by income, size, and resource access. Targeted policy interventions that empower women and address gender‐based inequalities in resource access and decision‐making are essential for improving food security and nutrition outcomes, particularly for female‐headed households.

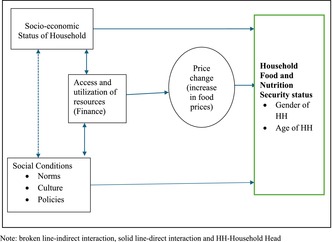

## 1 Introduction

Rising prices disproportionately impact low‐income individuals, as they are more vulnerable to changes in food prices due to their limited resources and the significant portion of their income allocated to food consumption (Degye et al. 2022). Perhaps the increasing cost associated with buying certain food items can force people to opt for cheaper, less healthy products that are more affordable and provide fewer nutritional benefits. Changes in food prices lead to both short‐term hardships and chronic malnourishment, which can have serious implications for the health and well‐being of future generations (Ayinde et al. 2012; Shittu et al. 2017). For example, Robles et al. (2010) showed that the price surge in 2007/2008 significantly decreased calorie consumption at both the national and household levels throughout Latin America, particularly affecting children under 2 years old from low‐income families. Consistently, other empirical studies (e.g., Arndt et al. 2016) documented that food price changes have a significant effect on children's growth and development.

Food price changes are closely related to the four pillars of food security: availability, accessibility, utilization, and stability (Amolegbe et al. 2021). Although an adequate food supply and household purchasing power are crucial, they do not guarantee food security. The cornerstone of individual well‐being is the ability to meet dietary needs, encompassing both macronutrient and micronutrient intake, as well as individual preferences. This dimension of utilization is influenced by factors such as intra‐household food distribution, cultural norms, and individual health conditions. Despite its critical role, the effective measurement of utilization is complex and resource‐intensive. This highlights the challenge of relying solely on indicators that primarily assess food availability, such as per capita calorie supply, or accessibility, such as income poverty.

Scholarly literature consistently demonstrates that the impact of food price fluctuations on household food security can vary significantly depending on a range of factors (Amolegbe et al. 2021). Notably, household income plays a crucial role, as evidenced by Engel's law, which posits an inverse relationship between income and the proportion of income spent on food. This implies that rising food prices disproportionately affect low‐income households. Furthermore, the impact depends on a household's position in the market. Net‐selling households, which produce and sell a particular food item, generally benefit from price increases. Conversely, net‐buying households that rely on purchasing food experience negative consequences. However, this dynamic is not always straightforward because household market roles can fluctuate seasonally. Empirical research further underscores the nuanced and complex relationship between food prices and household welfare. Arndt et al. (2016) and Shittu et al. (2015) highlight that the welfare implications of food price shocks are not uniform. They can result in either gains or losses, depending on the speed and extent to which labor and commodity markets respond to changes in food prices. For example, Shittu et al. (2015) found that agricultural households in rural Nigeria benefit from rising food prices. In contrast, Degye et al. (2022) documented that increased food prices led to a redistribution of wealth favoring higher‐income households, emphasizing the diverse and context‐specific nature of these impacts on food demand.

While the precise relationship between price fluctuations and food security outcomes remains debatable, the significance of price data in assessing food security risks cannot be understated. First, price movements are intimately connected to several key determinants of food security, including supply and demand dynamics, real income levels, and intermarket relationships. Second, compared to many other food security indicators, price data are readily available and relatively inexpensive to collect. Finally, prices effectively reflect the collective expectations and perceptions of a vast network of market participants regarding future supply and demand conditions and associated risks (Amolegbe et al. 2021). This inherent capacity of prices to distill and disseminate market information makes them valuable tools for researchers and policymakers. Understanding how household food demand responds to price shocks is crucial for accurately assessing their impact on household food and nutrition security. This knowledge is essential for developing effective interventions to mitigate the negative consequences of price volatility and ensure equitable access to food.

The food security impacts of rising food prices vary significantly across demographic and socio‐economic groups. For example, Kumar and Quisumbing (2013) found that female‐headed households in Ethiopia are more vulnerable to food price shocks, largely due to limited resources and wider food gaps compared to male‐headed households. These households often cope by reducing meal frequency, even during better months, and by relying on less preferred foods. Similarly, Jabbour et al. (2023) highlighted that women, particularly in the Eastern Mediterranean region, are disproportionately affected by rising food prices. This is attributed to their prioritization of household needs over their own well‐being, leading to higher rates of food insecurity, undernutrition, micronutrient deficiencies, and obesity among females than among men. Empirical evidence also indicates that rising food prices have a disproportionate impact on food security among children compared with adults. Studies by Bork and Diallo (2017) and Tumilowicz et al. (2015) revealed gender‐based disparities in stunting prevalence in Senegal and Guatemala, highlighting children's heightened vulnerability to nutritional deficiencies. Elevated food prices often result in reduced diet quality, contributing not only to undernutrition and micronutrient deficiencies but also to an increased risk of overweight and obesity among children. In low‐income households, even less nutritious foods may become unaffordable as prices escalate. Hirvonen et al. (2017) document the widespread nature of chronic undernutrition in Ethiopia, where many children consume monotonous and nutrient‐poor diets. This poses serious health risks, especially to lactating women and young children. In light of this, the present study underscores the need to assess dietary quality through the lens of overall dietary balance and diversity, rather than focusing narrowly on individual food groups.

In Ethiopia, the wholesale and retail prices of most cereals continuously increase. For instance, the price of maize per 100 kg increased from less than 500 birr in 2010 to about 3000 birr in 2022 (six‐fold), while the price of teff increased from about 800 birr per 100 kg to over 5000 birr (more than six‐fold) over the same period (Zewdie 2025). In recent years, significant price increases have been observed for major staple crops. For example, the price of five liters of edible oil increased by 50% compared to 600 birr in 2021/2022 and reached ETB 980 at retail prices in 2022. With an average low‐income family earning approximately ETB 2000 per month, this price increase equates to roughly half of their net income. Such substantial expenditure on a single food item severely limits these families' ability to purchase other essential goods and services, including other food items. This stark reality underscores the critical role of the price instability of staple foods in exacerbating food insecurity and poverty within the country.

Prior research investigating the impact of food price fluctuations on household food and nutrition security often overlooked significant variations among households (Amolegbe et al. 2021; Degye et al. 2022; Shittu et al. 2015). Some studies primarily examined the broader effects of price changes on household food security, while others focused on the determinants of malnutrition, emphasizing factors such as income, dietary practices, and access to sanitation and clean water. Only a few studies (Abebe and Delelegn 2013; Matz et al. 2015) have examined the effect of food price changes on household food security in Ethiopia.

To address this gap, this study investigates the impact of food price changes on household food security and diet quality while simultaneously examining potential gender disparities in these impacts. To achieve this, the study employed random effect and ordered random effect probit models. Furthermore, to quantify the gender gap in food and nutrition security due to food price changes, this study utilized the extended Kitagawa‐Oaxaca‐Blinder decomposition approach following Kröger and Hartmann (2021).

## 2 Conceptual Framework

When food prices increase, households often find themselves at the forefront of adjustment, recalibrating their expenditure patterns to navigate shifting terrain of affordability and accessibility. As food prices increase, households instinctively react by amplifying the share of their expenditure on food. This response stems from the primal instinct of ensuring sustenance and nourishment for oneself and family members amid the escalating cost of necessities. In practical terms, this means that a greater proportion of the household budget is channeled toward food purchases as families strive to safeguard against hunger and malnutrition. This leads to contractions in non‐food spending, such as education and healthcare, which have long‐term implications for investments in human capital, health outcomes, and overall well‐being.

Furthermore, the burden of these adjustments is not uniformly borne across all household members. Vulnerable segments, such as children, the elderly, and women, often bear the brunt of austerity measures, facing heightened risks of nutritional deficiency, diminished access to healthcare, and restricted educational opportunities. In contexts where gender norms dictate differential access to resources and decision‐making power, women may find themselves disproportionately affected, shouldering the responsibility of stretching limited resources to meet the needs of their families.

**FIGURE 1:**
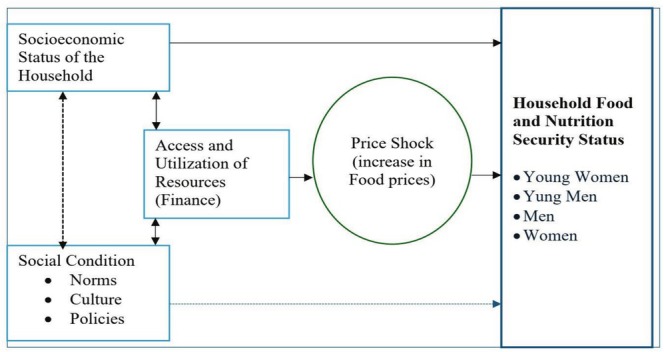
Disaggregating food and nutrition security by gender.

To understand and address this gender gap, we employed a gender analysis framework (given in Figure 1). A gender analysis framework is a tool used to assess how gender roles, norms, and relations influence various aspects of households, society, policies, and programs. It aims to understand and address gender disparities and promote gender equality (Maloiy and Wawire 2021). The Harvard, Moser (triple roles), and Equality and Empowerment (Longwe) frameworks are the most common gender analytical tools employed to address different aspects of gender disparities. The Harvard Analysis Framework is used to analyze the division of labor between men and women. Accordingly, this framework emphasizes the role of men and women in controlling and owning resources and their stake in defining the household’s objective function. Moreover, their role in making adjustments during shocks/changes defines the extent of the welfare loss or gain of a household.

It is not only the socioeconomic characteristics of a household but also the economic challenges women face in society that determine the magnitude of the effect of food price changes on household food security. Specifically, in developing countries, where women experience significant economic challenges in terms of access to resources and the right to family resources (Maloiy 2018), such gender disparity is inevitable. When it comes to food and nutrition security, the disparity goes beyond the household and impacts children, young boys, and girls. Therefore, this study will use the Harvard Analytical framework to disaggregate how food price changes impact the food and nutrition security status of the women‐headed and male‐headed households where gender is further disaggregated as young women (YW), young men (YM), men (M) and women (W). This conceptual framework is illustrated in Figure 1.

## 3 Methods and Data

### 3.1 Data Sources

This study utilized data from the Ethiopian Living Standards Measurement Survey (LSMS), which reached its fifth wave in 2011/2012. We focused on the two most recent waves (waves 4 [2018/2019] and 5, [2021/2022]), as earlier rounds of the survey could not be effectively integrated with the latest datasets. The LSMS is a comprehensive survey that covers various topics, including income and expenditure, employment, health and nutrition, and education.

For our analysis, we focused on household food consumption behaviors, consumption data, and household characteristics. Using this information, we computed several food and nutrition security indicators, including the Household Dietary Diversity Score (HDDS), the Food Insecurity Experience Scale (FIES), and household food expenditure shares.

Since the LSMS data do not include food price information, we supplemented our analysis with region‐specific food price data obtained from the Ethiopian Statistical Service (ESS) for the corresponding survey periods.

### 3.2 Nutrition and Food Security Indicators

#### 3.2.1 Household Dietary Diversity Score (HDD)

The HDDS, which reflects the dietary quality of a household, is an important indicator of a healthy diet. This indicator is crucial for understanding how dietary diversity responds to fluctuations in food prices. The HDDS is an indicator of household food access. For several reasons, the HDDS is an important indicator of food quality as well as food security, as it shows how many different food kinds a household consumes over a given reference period (INDDEX Project 2018).

We computed the HDDS following the standard FAO (2013) approach. First, we grouped the food items covered in the LSMS survey into 12 groups, following the food group definitions in FAO (2013). Each group was assigned a score of 1 (if consumed in the previous 24 h) or 0 (if not consumed in the last 24 h). The household score ranges from 0 to 12 and is equal to the total number of food groups consumed by the household during the last 24 h prior to the survey period. This is given by

(1) $${\text{HDDS}}_{j}=\sum_{i=1}^{12}w_{i}f_{j}$$

where ${\text{HDDS}}_{j}$ is the household diet diversity score for household j, i represents the number of food groups consumed by household over the last 24 h and i=1,2,…12; $w_{i}$ stands for the weight assigned to each food group (equal to one in this case); and $f_{j}$ is the different food groups consumed by the household j over the reference period (24 h).

Finally, following Kundu et al. (2020), we categorized the HDDS into three groups: households with HDDS dietary diversity scores of (0–3) as low, 4–6 as medium dietary diversity (4–6), and 7–12 as high dietary diversity (7–12).

#### 3.2.2 Food Insecurity Experience Scale (FIES)

The FIES is an experience‐based household food security measure derived from responses to eight standardized questions assessing access to adequate food over the past year. The questions capture self‐reported experiences related to resource constraints, such as worrying about food, inability to afford nutritious meals, reduced dietary variety, skipping meals, and running out of food. These questions can be easily integrated into population‐based surveys. Table 1 presents the eight items and their corresponding severity levels of food insecurity.

**TABLE 1:** Dimensions of food insecurity experience scale (FIES).

Item	Questions	Label	Assumed severity of FI
1	You were worried you would not have enough food to eat?	WORRIED	Mild
2	You were unable to eat healthy and nutritious food?	HEALTHY	Mild
3	You ate only a few kinds of foods?	FEWFOOD	Mild
4	You had to skip a meal?	SKIPPED	Moderate
5	You ate less than you thought you should?	ATELESS	Moderate
6	Your household ran out of food?	RUNOUT	Moderate
7	You were hungry but did not eat?	HUNGRY	Severe
8	You went without eating for a whole day?	WHLDAY	Severe

*Note:* During the last 12 months, was there a time when, because of lack of money or other resources?

*Source:* FAO (2017).

Based on responses to the food insecurity questions, we constructed a composite score to reflect the severity of food insecurity at the household‐level. The eight questions and their corresponding severity levels are presented in Table 1. Accordingly, households answering ‘yes’ to questions 1–3 are classified as experiencing mild food insecurity; those responding ‘yes’ to questions 4–6 as moderate; and those answering ‘yes’ to questions 7 and 8 as experiencing severe food insecurity.

#### 3.2.3 Percentage of Household Expenditure on Food (Food Share)

The share of household spending allocated to food reflects the household's susceptibility to food insecurity. This metric aids in understanding how households adapt to food price fluctuations. At the household level, this indicator is represented as the proportion of total expenditure that goes towards food. To derive this measure, the daily food spending of each household must be compared with their overall expenditures. Smith and Subandoro (2007) suggested that households dedicating over 75% of their income to food are at risk of food deprivation. This is because, regardless of their current food intake, a decrease in income would likely lead to reduced food consumption or a decline in food quality. Therefore, food share was calculated as follows:

(2) $$\text{Percentage of expenditure}\;\mathrm{on}\;\text{food}\;\left({\mathrm{FS}}_{j}\right)=\frac{\text{expenditure}\;\mathrm{on}\;\text{food}}{\text{total expenditure}}*100$$

Following Smith and Subandoro (2007), food share scores equal to or greater than 75%, 65%–75%, 50%–65%, and less than 50% were labeled as very high, high, medium, and low vulnerability to food deprivation, respectively. Table 2 presents a detailed description of the variables used in the analysis, along with their respective data sources.

**TABLE 2:** Variable descriptions and data sources.

Variable	Description	Data source
HDDS	Household dietary diversity score	LSMS
Food share	Percentage of household total expenditure allocated to food	LSMS
FIES	Food insecurity experience scale	LSMS
Food price	Average price of food items specific to regions of Ethiopia	ESS
Age	Age of household head categorized into three^a^	LSMS
Gender	Gender of household head	LSMS
Education	Education of household head classified into five groups^b^	LSMS
Marital status	Marital status of the household head (single, married, separated/widowed)	LSMS
Household size	Household size	LSMS
Place of residence	Place of residence (rural vs. urban)	LSMS
Employment status	Employment status of the household head (employed vs. unemployed)	LSMS

^a^ To classify age groups, we adapted the Ethiopian Statistical Service (EES) labor force categorization to better fit our data distribution. The original categories (less than 15, 15–19, …, above 60) were too granular, and enough observation was not found in each category. So, we reclassified them into three broader groups: less than 25, 26–49, and 50+.

^b^ We grouped the highest educational attainment of household head into five as illiterate, basic education, primary, secondary, Diploma/certificate, BA/BSc and MSc and above.

### 3.3 Estimation Strategies

To address the study's objectives, we employed various techniques to disentangle the effects of food price changes on households' consumption of healthy diets and their food security. The gender of the household head plays a crucial role in this analysis; thus, we estimated its implications for healthy diets and food security status. Additionally, the impact of food price changes on the affordability of nutritious diets and food security may differ between female‐headed and male‐headed households. Consequently, our econometric analysis focused on examining gender gaps in food security and nutritious diets with respect to food price changes.

To examine the effect of food price changes on household food security and nutritional status, we treated our indicators in two ways: continuous and binary variables. Our first two indicators, HDDS and food share, are continuous by nature, whereas the Food Insecurity Experience Scale (FIES) is a categorical variable. For the HDDS and food share, we estimated linear and non‐linear models for the panel data. For the FIES, we estimated a non‐linear panel data model given the categorical nature of the variable.

Thus, following Cameron and Trivedi (2005), the linear panel model was specified for the continuous versions of food share and HDDS,

(3) $$Y_{\mathrm{it}}=\alpha_{\mathrm{it}}+X_{\mathrm{it}}\beta_{\mathrm{it}}+u_{\mathrm{it}}$$

where i=1,2,…N and t=1,2,…T. $Y_{\mathrm{it}}$ is the food security indicator (HDDS and food share) for the household i at t, $X_{\mathrm{it}}$ is a vector of explanatory variables that includes food price, gender of household head, age of household head, place of residence (rural vs. urban), marital status, etc., $u_{\mathrm{it}}$ is the error term.

Equation (3) presents a model that helps us investigate the effect of food price changes on the household healthy diet status measured in terms of HDDS, as well as the effect of food price on household food security measured by food share.

In panel data analysis, where multiple measurements over time for the same individuals or entities are observed, unobserved heterogeneity factors that affect the dependent variable but are not directly observed are often encountered. To account for this heterogeneity, we employed different modeling techniques, including fixed effects, random effects, correlated random effects models, and conducted robustness checks using alternative definitions of our food and nutrition security indicators.

The fixed effect model (FE) assumes that unobserved heterogeneity is specific to each individual and is correlated with independent variables. The FE model removes the individual‐specific effects by taking the first difference or within‐group deviations. This effectively controls for time‐invariant unobserved heterogeneity. The random effect model (RE), on the other hand, assumes the unobserved heterogeneity is random and uncorrelated with the independent variables. RE assumes that the individual‐specific effects are random draws from a common distribution. The RE model is more efficient than the fixed effects model when the assumption of independence holds.

The other alternative model is the correlated random effects Model (CRE), which combines both the properties of FE and RE models. CRE assumes that the unobserved heterogeneity is correlated with some of the time‐invariant independent variables, and its model relaxes the assumption of independence between the individual‐specific effects and the time‐invariant regressors. This allows for a correlation between the two, making it a more flexible approach.

Thus, to make the right model decision, we have conducted the Hausman and Mundlak tests. The Hausman test compares the fixed and random effects estimators, and if the null hypothesis (random effects are consistent) is rejected, the fixed effects model is preferred. On the other hand, Mundlak tests whether the individual effects are correlated with the time‐invariant regressors, and if the null hypothesis (no correlation) is rejected, the correlated random effects model is preferred.

Furthermore, as discussed above, we transformed the continuous food security indicators into ordered categorical variables and estimated them using a random effects ordered probit model. The ordered random effects model is specified as

(4) $$\mathrm{Pr}\left(Y_{\mathrm{it}}>k|k,X_{\mathrm{it}},v_{i}\right)=\Phi \left(X_{\mathrm{it}}\beta +v_{i}-k_{k}\right)$$

where i=1,…n panels, t=1,…T, $n_{i},v_{i}$ are independently and identically distributed ($N0,{\sigma_{v}}^{2}$) and k is a set of cutpoints $k_{1},k_{2,}\ldots k_{K-1}$ where K is the number of possible outcomes and $\Phi \left(.\right)$ is standard normal cumulative distribution function.

From the above, we can derive the probability of observing the outcome k for response $Y_{\mathrm{it}}$ as

(5) Pitk=PrYit=kk,Xit,vi=Prkk−1<Xitβ+ϵit≤kk=Prkk−1−Xitβ−vi<ϵit≤kk−Xitβ−vi=Φkk−Xitβ−vi−Φkk−1−Xitβ−vi

where $k_{0}$ is taken as −∞ and $k_{k}$ is taken +∞. Here $X_{\mathrm{it}}$ does not contain a constant term because its effect is absorbed into the cutpoints.

In line with previous empirical studies like Amolegbe et al. (2021), we included a range of control variables: food price, age of the household head, household size, place of residence (urban vs. rural), marital status, employment status (employed vs. unemployed), and education level of the household head.

To further analyze the data, we employed the extended Kitagawa‐Oaxaca‐Blinder decomposition approach (Kröger and Hartmann 2021) on the household food share variable in its continuous form to decompose household food security indicator scores based on the gender of the household head. Following Kroger and Hartmann's (Kröger and Hartmann 2021) methodology, we examined the roles of the endowment effect and the coefficients of the included variables in explaining the gender gap in food security over time using Equation (6):

(6) $${\mathrm{Fsh}}_{t}^{l}=X_{t}^{l}\beta_{t}^{l}+u^{l}+\epsilon^{l},E\left(\epsilon^{l}\right)=0,\mathrm{cov}\left(X_{t},\varepsilon_{t}\right)=0,l\in \left[M,F\right]$$

where, Fsh represents household food share, *X* is a matrix encompassing the covariates, including the unity vector, while β comprises the *k* − 1 coefficients along with the constant term. The symbol *t* denotes time, *M* denotes male, *F* stands for female, and ϵ signifies the error term.

By utilizing the concept of group differences in coefficients, referred to as the threefold decomposition, we illustrate our notation decomposition in Equation (7)

$$\begin{array}{c} ∆{\mathrm{Fsh}}_{t}=E_{t}+C_{t}+I_{t}+U\\ E_{t}=\left\{E\left(X_{t}^{M}\right)-E\left(X_{t}^{F}\right)\right\}\beta_{t}^{F} \end{array}$$

(7) $$\begin{array}{c} C_{t}=E\left(X_{t}^{F}\right)\left(\beta_{t}^{M}-\beta_{t}^{F}\right)\\ I_{t}=\left\{E\left(X_{t}^{M}\right)-E\left(X_{t}^{F}\right)\right\}\left(\beta_{t}^{M}-\beta_{t}^{F}\right)\\ U=\left\{E\left(u^{M}\right)-E\left(u^{F}\right)\right\} \end{array}$$

where $E_{t}$ represents the portion of the difference attributable to variations in the groups' characteristics at time t, known as the endowment effect. $C_{t}$ accounts for part of the difference resulting from disparities in the coefficients at time, *t*. Furthermore, $I_{t}$ signifies the segment of the difference at time *t* that arises from the interaction between the groups' differing characteristics and coefficients. Finally, U captures part of group differences that are attributed to unobserved factors that do not change within the observation period.

## 4 Results and Discussion

This section offers a comprehensive view of the multifaceted impacts of food price changes on household food security and nutrition status. For this, we used different descriptive and econometric techniques and disaggregated the results by the age and gender of the household head.

### 4.1 Price Change and Household Food Security

#### 4.1.1 Share of Food Expenditure

The response of households to changes in food prices illuminate the intricate interplay between economic forces and human resilience. To illustrate this dynamic in the Ethiopian context, Table 3 presents a comparison of food expenditures and food prices across two periods, 2018/2019 and 2021/2022.

**TABLE 3:** Share food expenditure.

Year	Food	Expenditure	Food	Price	Observation
	Mean	Std. Dev.	Mean	Std. Dev.	
2018/2019	0.71	0.20	20.33	2.96	1921
2021/2022	0.73	0.17	31.83	4.93	4959
Difference	0.02***	0.003	11.5***	0.07	6880

*** Statistically significant at 1%, def = mean (2021/2022)‐mean (2018/2019).

*Source:* Authors' computation based on LSMS (2018/2019–2021/2022).

Between 2018/2019 and 2021/2022, Ethiopian households experienced a rise in both the mean food expenditure share, from 0.71 to 0.73, and average food prices, which increased markedly from 20.33 to 31.83. This can be attributed to multiple underlying factors, with food price inflation is a primary driver. A growing imbalance between the domestic food supply and the rising demand of an expanding population has exerted upward pressure on prices (Zewdie 2025). Structural inefficiencies in food supply chains, particularly excessive mark‐ups by intermediaries, have escalated prices. Moreover, agricultural productivity has been adversely affected by armed conflicts, internal displacement, the COVID‐19 pandemic, extreme weather events, and locust invasions, mainly in the last few years. The war in Ukraine has exacerbated these challenges by disrupting global supply chains and driving up the costs of imported food commodities, petroleum products, and fertilizers.

Food prices also vary significantly by commodity. Gebremeskel (2020) identified the top five commodities with the highest average inflation rates between 2017 and 2020: onions (17%), barley (13%), garlic (12%), bread (9%), and chickpea (7%). These figures suggest that both perishable goods and staple food items are particularly vulnerable to price changes, reflecting supply constraints, seasonal production patterns, and market inefficiencies.

This trend reflects growing economic pressure, as households allocate a larger portion of their income to food to meet their basic needs. As shown in Table 4, the proportion of households in the “very high” food share category, those allocating 75% or more of their expenditure to food, increased slightly from 51% in 2018/2019 to 52% in 2021/2022. Similarly, the shares of households in the “high” (65%–75%) and “medium” (50%–65%) deprivation categories rose from 19% to 20% and from 17% to 18%, respectively. Most concerning is the decline in households classified under the “low” deprivation category (food share < 50%), which fell from 12% to 10% over the same period. These shifts indicate a gradual deterioration in household food security, with more families slipping into higher deprivation categories and fewer families maintaining relatively secure levels of food access.

**TABLE 4:** Household food deprivation.

Food deprivation scale	Year			Diff
	2018/2019 (%)	2021/2022 (%)	Total (%)	(%)
Very high	51	52	52	0
High	19	20	20	2**
Medium	17	18	18	1
Low	12	10	11	−3***

*Note:* Pearson chi^2^(3) = 21.5221 Pr = 0.000. *** *p* < 0.01 and ** *p* < 0.05.

*Source:* Authors' computation based on LSMS (2018/2019–2021/2022).

As reported in Table 4, the proportion of households in the very high food deprivation category increased slightly from 51% in 2018/2019 to 52% in 2021/2022, while the percentage of households facing high deprivation increased from 19% to 20% and medium deprivation from 17% to 18%. This suggests a persistent and possibly growing struggle for a significant portion of the population to access adequate food supply. Perhaps most worrying is the decline in households experiencing low food deprivation, which dropped from 12% in 2018/2019 to just 10% in 2021/2022. This implies that households that were previously in a relatively better food security situation are slipping into more severe deprivation categories.

#### 4.1.2 Household Food Insecurity Experience Scale (FIES)

Table 5 reveals a significant shift in Ethiopia's food security situation between 2018/2019 and 2021/2022, as measured by the FIES. In 2018/2019, nearly half (49.61%) of the households experienced mild food insecurity. This indicates that they faced some challenges in accessing adequate food but likely did not skip meals or experience significant dietary limitations. Another significant portion (44.02%) dealt with moderate food insecurity, suggesting that they had to make sacrifices, such as reducing food variety or quantity and shifting to less healthy and nutritious food. Only a small percentage (6.37%) faced severe food insecurity, meaning that they struggled to obtain enough food and likely went hungry at times. The picture in 2021 is concerning. There were dramatic decreases in the percentages of households experiencing both mild and moderate food insecurity (down to 29.73% and 24.28%, respectively). The significant decline in the “better‐off” categories (mild and moderate) coincides with a worrying increase in the percentage of households facing severe food insecurity. This number skyrocketed from 6.37% in 2018/2019 to a staggering 45.99% in 2021/2022. This means that nearly half of all households surveyed in 2021/2022 were severely food insecure, struggling immensely to access enough food.

**TABLE 5:** Household food insecurity experience scale.

FIES categories	2018/2019 (%)	2021/2022 (%)	Change (%)
Mild	49.61	29.73	−19.88
Moderate	44.02	24.28	−19.74
Severe	6.37	45.99	39.62
Observation	518	2916	—

*Note:* Pearson chi^2^(2) = 878.7240 Pr = 0.000.

*Source:* Authors' computation based on LSMS (2018/2019–2021/2022).

Overall, the results reveal a grim picture of worsening food security in Ethiopia. While there may be a decrease in those experiencing milder forms of food insecurity, it is overshadowed by a significant rise in the number of households facing severe hunger. This data suggests a critical situation for many Ethiopians and the need for urgent interventions to address this growing food insecurity.

Table 6 highlights the gender gap in food security between 2018/2019 and 2021/2022 as measured by household FIES. In 2018/2019, while more than half of households headed by males (52.4%) experienced mild food insecurity, a slightly lower proportion (45.37%) of female‐headed households faced the same challenge. This trend continued in moderate food insecurity, with a higher percentage of female‐headed households (47.8%) than male‐headed ones (41.53%). Notably, the percentage of households experiencing severe food insecurity was relatively similar for both genders (approximately 6%). However, the situation in 2021/2022 showed a significant deterioration. There were substantial decreases in the percentages of both sexes experiencing mild and moderate food insecurity. While the proportion of males experiencing mild food insecurity dropped by 22.17 percentage points, for females, the decline was slightly less severe (16.69 percentage points). Similarly, the decrease in moderate food insecurity was steeper for females (26.85%) than males (15.69%).

**TABLE 6:** FIES by gender.

FIES	2018/2019		2021/2022		Change	
	Male	Female	Male	Female	Male	Female
Mild	52.4	45.37	30.23	28.68	−22.17	−16.69
Moderate	41.53	47.8	25.84	20.95	−15.69	−26.85
Severe	6.07	6.83	43.93	50.38	37.86	43.55
Observation	313	205	19,985	931	—	—

*Note:* Pearson chi^2^(2) = 521.4442 Pr = 0.000 for males and Pearson chi^2^(2) = 521.4442 Pr = 0.000 for females.

*Source:* Authors' computation based on LSMS (2018/2019–2021/2022).

The concerning aspect is the dramatic increase in severe food insecurity, particularly among female‐headed households. The percentage of females in this category increased from 6.83% in 2018/2019 to a staggering 50.38% in 2021/2022, representing a nearly eight‐fold increase. Even for male‐headed households, there was a substantial rise (from 6.07% to 43.93%). We also disaggregated the FIES data by age group.

In terms of age group, Table 7 shows that a significant increase in severe food insecurity across all age groups. While the increment is noticeable across all age brackets, a particularly dramatic surge was observed within the second age group, encompassing individuals aged 26–49 years. However, it is noteworthy that in 2021/2022, the youngest age group, comprising individuals less than 25 years old, exhibited the highest percentage of households experiencing severe food insecurity. This indicates a concerning trend wherein younger demographics are disproportionately affected by food insecurity despite the overall upward trend across all age groups.

**TABLE 7:** FIES by age group.

Age group (AG)^a^	FIES‐2018/2019			FIES‐2021			Change		
	Mild (%)	Moderate (%)	Severe (%)	Mild (%)	Moderate (%)	Severe (%)	Mild (%)	Moderate (%)	Severe (%)
AG1	62	29	9	27	29	45	−35	−1	35
AG2	50	44	5	30	25	45	−21	−19	40
AG3	42	52	6	30	23	47	−12	−29	41

*Note:* Pearson chi^2^(2) = 23.9105 Pr = 0.000 for AG1; Pearson chi^2^(2) = 183.5492 Pr = 0.000 for AG2; and Pearson chi^2^(2) = 162.6578 Pr = 0.000 for AG3.

^a^ Age Group definition: AG1: < 25 years, AG2: 26 to 49, and AG3: above 50+.

*Source:* Authors' computation based on LSMS (2018/2019–2021/2022).

### 4.2 Food Price and Household Diet Diversity

As discussed in Section 3.2, the HDDS measures a household's dietary quality. We used this indicator to analyze how household dietary diversity responds to fluctuations in food prices.

As reported in Table 8, in 2018/2019, a notable proportion of Ethiopian households (18.21%) attained a very high HDDS. This suggests that these households maintained a diet characterized by a diverse array of food groups, reflectiving a balanced and nutritious dietary intake. Moreover, a substantial majority, encompassing 59.75% of households, achieved a high HDDS, further reinforcing the notion of a generally adequate diet in the sampled population. However, approximately 22% of households fell within the low HDDS category, which is concerning. This classification signals a potential vulnerability to nutrient deficiencies and food insecurity among this segment of the population. In 2021/2022, there was a significant decline in the proportion of households with very high and high HDDS. The percentage of households with a very high HDDS plummeted to a mere 0.29%, and those with a high HDDS dropped significantly to 15.85%. Conversely, there has been a sharp rise in the proportion of households with a low HDDS, with an approximately 84% of households falling into this category, representing a substantial increase of 62% compared to the figure in 2018/2019. This significant shift suggests a potential deterioration in dietary diversity in Ethiopian households.

**TABLE 8:** HDDS category by year.

HDDS category	2018/2019 (%)	2021/2022 (%)	Change (%)
Very high	18.21	0.29	−17.92
High	59.75	15.85	−43.9
Low	22.05	83.87	61.82

*Note:* Pearson chi^2^(2) = 4.1e+03 Pr = 0.000.

*Source:* Authors' computation based on LSMS (2018/2019–2021/2022).

The implication is that a large number of households have transitioned from consuming a more balanced and nutritious diet (very high and high HDDS) to a less balanced and less nutritious diet (low HDDS). This shift raises concerns about potential nutrient deficiencies and increased food and nutrition insecurity among Ethiopian households.

While the overall trend in Table 8 indicate a decline in dietary diversity across Ethiopian households, a closer look at the data by gender (Table 9), reveals some interesting variations. Both male and female‐headed households experienced a significant decrease in the very high and high HDDS categories between 2018/2019 and 2021/2022. This suggests a general decline in the consumption of a balanced and nutritious diet. However, the extent of this decline appears to be slightly different between genders. Female‐headed households seem to have been disproportionately affected, experiencing a relatively larger decline in the high HDDS category compared to male‐headed households. Conversely, the increase in the low HDDS category, indicating a less balanced diet, was also slightly higher for female‐headed households. This suggests that female‐headed households may be facing greater challenges in securing access to a diverse and nutritious diet compared to their male counterparts. It is possible that factors like economic hardship, limited access to resources, or social inequalities could play a role.

**TABLE 9:** HDDS by gender (2018/2019–2021/2022).

HDDS category	2018/2019			2021/2022			Change		
	Mild (%)	Moderate (%)	Severe (%)	Mild (%)	Moderate (%)	Severe (%)	Mild (%)	Moderate (%)	Severe (%)
AG1	62	29	9	27	29	45	−35	−1	35
AG2	50	44	5	30	25	45	−21	−19	40
AG3	42	52	6	30	23	47	−12	−29	41

*Note:* Pearson chi^2^(2) = 2.8e+03 Pr = 0.000, male Pearson chi^2^(2) = 1.3e+03 Pr = 0.000, female.

*Source:* Authors' computation based on LSMS (2018/2019–2021/2022).

In terms of age groups, a general decline in dietary diversity was observed across all age groups between 2018/2019 and 2021/2022. However, a more pronounced decrease was observed in the second age group. This was particularly observed in the ‘Very High HDDS’ category, suggesting a potential shift away from highly diversified, nutrient‐rich diets among this age group. Concurrently, a relative increase in the ‘Low HDDS' category was observed for this age group, indicating a trend towards less balanced dietary patterns. Life stage transitions, such as family formation or career changes, may influence dietary habits, access to diverse and nutritious foods, and household budgetary constraints, potentially contributing to this specific trend among the second age group (Table 10).

**TABLE 10:** HDDS category by age group and year.

HDDS category	Age group (2018/2019)			Age group (2021/2022)			Age group (change)		
	Mild (%)	Moderate (%)	Severe (%)	Mild (%)	Moderate (%)	Severe (%)	Mild (%)	Moderate (%)	Severe (%)
AG1	62	29	9	27	29	45	−35	−1	35
AG2	50	44	5	30	25	45	−21	−19	40
AG3	42	52	6	30	23	47	−12	−29	41

*Note:* Pearson chi^2^(2) = 227.0001 Pr = 0.000, AG1; Pearson chi^2^(2) = 2.6e+03 Pr = 0.000 for AG2; and Pearson chi^2^(2) = 1.2e+03 Pr = 0.000 for AG3.

*Source:* Authors' computation based on LSMS (2018/2019–2021/2022).

### 4.3 Regression Results

#### 4.3.1 Model Selection Tests

The Hausman test, reported in the last rows of Table 11, indicates that we cannot reject the null hypothesis, suggesting that an RE model is appropriate for our analysis. Additionally, the intragroup correlation (*ρ*) for the RE model is close to zero for both models, further supporting the RE specification’s suitability. The overall model fit, as indicated by the F‐statistics, suggests that the coefficients of the included variables are jointly statistically significant, contributing to the explanatory power of the model. Furthermore, the Mundlak test, which examines the significance of the mean values of continuous variables as additional regressors, reveals that these mean values are not statistically different from zero. This finding reinforces the appropriateness of the RE model, as it suggests that the individual‐specific effects are not systematically related to the observed explanatory variables. Therefore, based on these diagnostic tests, we conclude that the RE model is the most suitable for our analysis. The following discussion is based on the results obtained from this model.

**TABLE 11:** The impact of price changes on household food share.

Variables	HDDS		Food share	
	Coefficient	Standard err	Coefficient	Stand err
Price	−0.188***	−0.00574	0.00320***	−0.00053
Household size	0.0662**	−0.0261	0.00342	−0.00244
Residence (Urban = 1)	1.191***	0.134	−0.141***	−0.0125
Gender (female = 1)	0.238**	−0.116	0.0452***	−0.0109
Empl (employed = 1)	1.027***	−274	−0.0251	−0.0256
*Education (base = illiterate)*				
Primary	−0.438	−0.596	−0.0161	−0.01
Secondary	0.235	−0.16	−0.0246	−0.0172
Certificate	0.0717	−0.203	0.0725**	−0.0292
BA/BSc	0.793***	−0.147	0.0771*	−0.0405
MSc and above	0.268	−0.187	−0.0813	−0.053
*Marital status (base = single)*				
Married			0.0975***	−0.0137
Separated			0.0794***	−0.0175
*Age (base category = Age group 1, less than 25 years)*				
Age group 2 (25–49)	0.304	−1.318	−0.0172	−0.0151
Age group 3 (above 49)	0.0717	0.203	−0.0132	−0.019
Constant	7.453***	1.29	0.721***	−0.121
Rho (*ρ*)	0.0095		0.0267	
Observations	1681		1681	
Prob > *F* = 0.000				
Hausman Test: *χ* ^2^(14) = 17.65, Prob > *χ* ^2^ = 0.2243				

*** *p <* 0.01.

** *p <* 0.05.

* *p <* 0.1.

*Source:* Authors' estimation based LSMS data (2018/2019–2021/2022).

#### 4.3.2 Food Price Changes, Food Share and Household Diet Diversity

The coefficient for food prices is positively and significantly associated with household food expenditure share and negatively with the HDDS, indicating that rising food prices increase the share of household budgets allocated to food while reducing diet diversity. Specifically, a 1% increase in food prices leads to a 0.32% rise in the food expenditure share and an 18% decline in the HDDS, suggesting that households reallocate spending towards staple or less nutritious foods to maintain consumption. These findings are consistent with those of Ayinde et al. (2012), Shittu et al. (2017), and Robles et al. (2010), who observed declines in calorie intake among vulnerable populations following food price hikes. Arndt et al. (2016) noted adverse effects on children's growth due to reduced access to nutritious foods, while Bai et al. (2021) emphasized the declining affordability of nutrient‐rich diets following an increase in food prices. Furthermore, Ruel et al. (2010) showed that food price shocks force poor households to prioritize food over other essentials, and Lele et al. (2016) found that higher food prices reduce household spending on health and education, threatening long‐term well‐being and human capital development.

Urban households exhibit lower food expenditure shares and higher diet diversity than rural households, as reflected by a strong positive coefficient in HDDS (1.191) and a negative coefficient in food share (−0.141). This can be attributed to greater market access, higher income and wealth levels, and broader food choices in urban settings, which foster more diverse diets. These findings align with those of Abu and Soom (2016) and Kundu et al. (2021), who report better food security and nutrition among urban populations. In contrast, Walsh and Van Rooyen (2015) found that rural households in South Africa's Free State Province had greater food availability, highlighting their role as net producers. Hadley et al. (2011) further emphasize that wealth buffers urban households from the adverse effects of rising food prices, although they tend to allocate a smaller share of income to food compared to rural households.

Female‐headed households exhibit higher food expenditure shares and better diet diversity (HDDS) than male‐headed households, likely due to gendered income allocation patterns and women's central role in household food management. Lower‐income female‐headed households may prioritize food spending to meet basic needs, contributing to a positive HDDS coefficient. This aligns with Botreau and Cohen (2019), who found women allocate a larger budget share to food, and with FAO (2008), which reported greater diet diversity in female‐headed households. Cultural norms often place women in charge of food procurement and preparation, positioning them as nutritional gatekeepers who prioritize health and well‐being (Ruel et al. 2010). Their attention to dietary preferences and use of social networks further enhances access to diverse and nutritious foods among female‐headed households.

Household size was positively associated with diet diversity, suggesting that larger households may benefit from pooled resources, diverse skills, and collective income‐generating activities that enhance purchasing power. While earlier studies (e.g., Akukwe 2020; Olayemi 2012) argue that larger households face food security challenges due to high dependency ratios, our findings align with Assefa and Abide (2023) and Pakravan‐Charvadeh et al. (2022), who report positive links between household size and food security. Larger families may also leverage social networks, accommodate varied dietary needs, and plan meals more efficiently, factors that collectively support greater dietary diversity and resilience to food system shocks.

The results revealed a strong and statistically significant positive association between employment status and dietary diversity, with a coefficient of 1.027. This indicates that households with employed family members are more likely to consume a wider variety of foods than households with no employed members. This relationship may be attributed to the higher disposable incomes of employed individuals, which enhance their ability to purchase diverse and nutritious foods. In addition, employment can offer indirect benefits through structured workplace nutrition programs and access to subsidized meals, further supporting dietary diversity. These findings are consistent with those of prior studies. Mengistu and Kassie (2022) reported that households without employment were more likely to be food insecure than those with at least one employed member. Similarly, Sankar et al. (2025) found a significant positive relationship between the number of employed individuals in a household and the overall food security status. Households with multiple employed members were notably more likely to achieve food security, suggesting that employment not only improves individual dietary outcomes but also contributes to household‐level resilience. These findings underscore the critical role of employment in shaping both access to and quality of food, reinforcing the importance of labor market interventions in addressing food and nutrition security. Education is also a significant determinant of dietary diversity. Individuals with a BA/BSc degree tended to report higher dietary diversity scores than those with only basic or no formal education. This finding is consistent with the literature (Samuel et al. 2020; Kundu et al. 2021), which highlights the role of educational attainment in improving nutritional awareness and food choice, thereby enhancing food security outcomes.

Marital status also plays an important role. Households headed by married individuals exhibit greater dietary diversity than those headed by single individuals. This may reflect shared household responsibilities, pooled income, and enhanced food preparation planning. Consistent with this finding, Mengistu and Kassie (2022) reported that households with married heads are more likely to achieve food security than those led by never‐married individuals.

Finally, age is a critical factor affecting food and nutrition security. Individuals aged 25–49 years demonstrated a significantly higher likelihood of consuming a diverse diet than those aged 25 years and younger. This result supports the descriptive analysis presented in Section 4 and may reflect greater economic stability, life experience, and awareness of nutritional needs within this age group.

### 4.4 Robustness Checks

To enhance the robustness and validity of the results presented above, we conducted additional checks using alternative definitions for food and nutrition security indicators. Specifically, the continuous dependent variables representing household food and nutrition security were transformed into corresponding categorical variables, in line with methodologies employed in similar studies. This approach allowed for the cross‐validation of findings and strengthened the credibility of the analysis. Specifically, the HDDS and food share were converted into categorical variables, while the FIES is a categorical variable by nature, as detailed in the descriptive section. The results from the random effects ordered probit model estimated using Equations (4) and (5) are reported in Table 12.

**TABLE 12:** Results of the ordered probit model.

Variables	(1)	(2)	(3)
	HDDS	FIES	Food share
Food price	0.0930***	0.0521***	−0.00649***
	(0.00228)	(0.00259)	(0.00159)
HH size	−0.0287***	0.0477***	−0.0204***
	(0.00563)	(0.00829)	(0.00621)
Residence (Urban = 1)	−0.753***	−0.101***	1.044***
	(0.0262)	(0.0359)	(0.0296)
Gender	0.0347	0.133***	−0.165***
	(0.0320)	(0.0479)	(0.0348)
*Marital status (base category = single)*			
Married	−0.146***	−0.0842	−0.491***
	(0.0464)	(0.0773)	(0.0490)
Separated	−0.0207	0.0482	−0.385***
	(0.0539)	(0.0860)	(0.0569)
*Age group (base category = Age group 1, less than 25 years)*			
Age group 2	0.0335	−0.0420	0.175***
	(0.0458)	(0.0771)	(0.0501)
Age group 3	−0.119**	−0.0471	0.107*
	(0.0496)	(0.0810)	(0.0546)
Cut1	−0.822**	1.489***	1.099***
	(0.352)	(0.527)	(0.371)
Cut2	0.810**	2.511***	1.767***
	(0.352)	(0.530)	(0.371)
*σ* ^2^	0.0527**	0.0947*	0.262***
	(0.0236)	(0.0512)	(0.0296)
Observations	11,684	4914	11,684
*χ* ^2^(1)	5.53	4.04	118.95
Prob ≥ *χ* ^2^	0.0093	0.0222	0.0000

*Note:* Figures in parentheses are standard errors.

*** *p <* 0.01.

** *p <* 0.05.

* *p <* 0.1.

*Source:* Authors' computation based on LSMS data (2018/2019–2021/2022).

Before interpreting the results, it is important to establish a common understanding of what each category represents in various food and nutrition security measures. For both HDDS and FIES, moving from one category to the next indicates a worsening household food or nutrition security. In contrast, for food share, transitioning from the first category to the next signifies an improvement as households move from a highly food insecure status to a relatively better status. Thus, moving from category one to category four reflects enhanced food security for this indicator (e.g., Kundu et al. 2021; Samuel et al. 2020).

The results in Table 12 indicate that the coefficients for food prices are positive and statistically significant for both HDDS (0.0930) and FIES (0.0521), while the coefficient for food share is negative (−0.00649). This indicates that as food prices rise, the likelihood of households experiencing a decline in their food security status increases. Specifically, for HDDS and FIES, higher food prices raise the probability that households will move from the first category (indicating better food security) to the next (which reflects severe food insecurity and less dietary diversity), confirming the result reported above. Conversely, the negative coefficient for food share suggests that as food prices increase, the likelihood of households transitioning from the first category (highly food insecure) to the next category (relatively food secure) decreases. This also confirms our result reported in Table 11, indicating that rising food prices not only restrict access to diverse food options but also exacerbate food insecurity, making it more challenging for households to improve their food security status.

Overall, the robustness check using alternative food and nutrition security indicators, as reported in Table 12, aligns with and strengthens the main findings in Table 11. These results underscore the adverse implications of food price inflation on household food security and dietary diversity, highlighting the urgent need for policy interventions to enhance food affordability and access.

Consistent with the results reported in Table 11, household size significantly influenced food security and dietary diversity outcomes. The negative coefficient for household size in the HDDS model indicates that larger households are more likely to maintain diverse diets, likely because of pooled resources and greater collective purchasing power. This is consistent with our main result obtained using the continuous version of the HDDS and reported in Table 11. Similar findings are obtained using food share, where household size reduces the probability that households move from the food insecure to the food secure category. However, the FIES, however, yields somewhat contrasting results, where large household size increases the probability of households moving to a severe food security status. This could be due to the difference in the focus and measurement of HDDS and FIES, where the focus of FIES is more on food security, while HDDS is more about dietary quality.

The results presented in Table 12 align with our main findings, indicating that urban households experience greater dietary diversity and lower food insecurity than their rural counterparts. This not only reinforces the robustness of our analysis but also underscores the heightened vulnerability of rural households to food and nutrition insecurity, particularly during periods of rising food prices. Similar patterns were observed for marital status and household age categories, further strengthening the results reported in Table 11. Additionally, the Likelihood Ratio (LR) test confirmed the overall significance of the model across all three outcomes (*p* < 0.05), demonstrating that the included variables provided meaningful explanatory power.

### 4.5 Decomposition Analysis

To investigate gender disparities in food security between female‐headed and male‐headed households, we employed the extended Oaxaca‐Blinder decomposition technique, as outlined in Kröger and Hartmann (2021). This extended methodology is particularly suitable for continuous variables. Consequently, in alignment with our model estimating food security based on food share, we decomposed the gender gap in food security utilizing a random effects model, which we have identified as our preferred approach for this analysis.

In the first part of Table 13, the “non‐parametric” row presents the mean group differences in food share among households, estimated non‐parametrically from the observed data. The subsequent rows in the “Decomp” section detail the decomposition of food share gaps into four distinct components: endowment, coefficient, and interaction effects, and the portion attributable to the time‐constant error term (random effects). The results reveal that the coefficient for the endowment effect is −0.054 in 2018/2019, contrasting with 0.394 in 2021/2022. This suggests that the observed variables included in our model, such as food prices, household size, urban versus rural residence, employment status (employed versus unemployed), age group, marital status, and regional dummies, played a minimal role in explaining the food share gap in 2018/2019. However, they began to positively influence this gap explaining 0.394% in 2021/2022. A significant portion of the gap in food share is attributed to the coefficients, with a coefficient of −3.058, which accounted for approximately 97% of the gap in 2018/2019 and an even higher 115% in 2021/2022. This indicates that while the observed characteristics of households became increasingly relevant in explaining the food share gap between female‐nd male‐headed households, the findings indicate a significant role of the coefficient in elucidating the gap in food share between female‐headed and male‐headed households while controlling for differences in endowments both between groups and over time.

**TABLE 13:** Decomposition results.

	2018/2019	2021/2022
Level non‐parametric‐c decomp	−3.146	−2.667
Endowments	−0.054	0.394
Coefficients	−3.058	−3.058
Interaction	0.000	0.000
RE	−0.034	−0.003
Total	−3.146	−2.667
Decomp %		
Endowments	1.705	−14.79
Coefficients	97.206	114.659
Interaction	0.000	0.000
RE	1.089	0.131
Total	100.000	100.000
Decomposition of change		
Change non‐parametric c	0.000	0.483
Decomp	0.000	0.448
Endowments	0.000	0.004
Coefficients	0.000	0.000
Interaction	0.000	0.000
RE	0.000	0.031
Total	0.000	0.483
Decomposition (%)		
Endowments		93.577
Coefficients		0.000
Interaction		0.000
RE		6.423
Total		100.000

Abbreviation: RE, random effects.

*Source:* Authors' estimation based on LSMS data (2018/2019–2021/2022).

Notably, the Random Effects (RE) component was found to be zero, suggesting that our model is reasonably well specified, and effectively captures the underlying dynamics influencing food share disparities.

The second part of Table 13 presents the results of the decomposition of the change in food share between 2018/2019 and 2021/2022. Notably, the gap in food shares increased by 0.5% during this period. Looking further into the roles played by shifting endowments and coefficients over time, the data reveal that the change in the endowment effect contributed an increase of a 0.4% to the food share gap, indicating that variations in household characteristics such as size, income, and demographic factors had a significant impact on food share disparities. In contrast, the contribution of changing coefficients was negligible, suggesting that shifts in how these characteristics influence food share did not substantially affect the gaps.

Additionally, the portion attributable to differences between groups in the time‐constant error term contributed to a 0.031% increase in the gap. Although this figure is relatively small, it highlights the importance of group‐specific panel dropout, suggesting that the dynamics of household participation in the study may subtly influence the observed results. Overall, these findings underscore the significance of endowment changes in driving food share disparities, while indicating that the structural relationships represented by the coefficients remain stable over time.

## 5 Conclusions and Policy Implications

This study delves into the complex connection between fluctuations in food prices and household food security in Ethiopia, utilizing a mixed‐methods framework that integrates quantitative data with qualitative insights. The findings revealed a complex interplay of factors influencing household food security, with food price changes emerging as a significant driver. Our empirical analysis indicates that escalating food prices substantially increase the proportion of household income allocated to food expenditures. This necessitates a reduction in non‐food spending, disproportionately affecting vulnerable groups such as female‐headed households (FHHs) and low‐income households, as they prioritize essential consumption over non‐essential consumption.

The impact of this is notably uneven. Urban households, benefiting from higher incomes and greater market access, exhibit lower food expenditure than their rural counterparts. Additionally, larger households, such as those that are married or separated, allocate a greater share of their income to food, underscoring the influences of income, household size, and access to resources.

Rising prices are critically associated with a decline in the HDDS. This reduction in dietary diversity is observable across various households, with a particularly notable impact on FHHs and individuals aged 26–49 years. Diminished purchasing power compels a transition towards less diverse and less nutritious diets, as financial constraints restrict available food choices. This study further examined the relationship between food price changes and household dietary diversity, measured using the HDDS. The results revealed a decline in dietary diversity across Ethiopian households, particularly among female‐headed households and those in the 26–49 age group. This decline is likely linked to rising food prices and reduced purchasing power, leading to a shift toward less diverse and nutritious food options. Intuitively, when food prices rise, households may opt for cheaper, less nutritious food items to stretch their budgets, compromising their dietary diversity and overall nutritional intakes.

Furthermore, the extended Oaxaca‐Blinder decomposition analysis revealed that while the endowment effect played a minimal role in explaining food share disparities in 2018/2019, its influence grew substantially in 2021/2022. However, the primary driver of the food share gap remains the coefficient effect, suggesting that household characteristics are a key factor influencing food share. This implies that interventions aimed at addressing the underlying structural inequalities and empowering female‐headed households to leverage their resources more effectively could be crucial in narrowing the food share gap.

The findings of this study underscore the critical importance of addressing food price changes and their consequences for household food security in Ethiopia. To successfully implement evidence‐based policies, both immediate disruptions and the underlying causes of vulnerability must be addressed. In the short term, policymakers should prioritize market stabilization and the protection of household purchasing power, focusing on vulnerable groups such as female‐headed households. Strengthening the Productive Safety Net Programme (PSNP) through complementary support measures such as income generating activities can enhance its effectiveness and responsiveness during periods of price surges. Expanding the weekend open markets currently operating in selected areas of Addis Ababa to other regions of the country would enable direct linkages between producers and consumers, thereby reducing reliance on intermediaries, who often drive up consumer prices.

Second, building long‐term resilience necessitates the inclusive transformation of the agricultural sector. Investments in rural infrastructure, including roads and storage facilities, are essential for reducing market barriers, lowering transaction costs, and minimizing post‐harvest losses. Furthermore, diversifying livelihoods through non‐agricultural opportunities, such as agro‐processing and rural enterprises, for FHHs and youth, facilitated by skills training and microfinance, is vital for reducing dependence on volatile food markets, mainly during shocks.

Third, expanding and strengthening the School Feeding Programme (SFP), particularly in rural areas, is critical for improving household food and nutrition security. By increasing children's access to food, the program also stimulates local economies through stronger linkages with local food supply chains. FHHs should be actively prioritized, with program design adaptations that address their specific constraints and vulnerabilities. Furthermore, addressing the structural inequalities highlighted by this analysis, especially the enduring coefficient effect, requires a concentrated effort to empower Ethiopian women. Ensuring and enforcing women's land rights, whether through joint or individual titles, and enhancing their access to credit, inputs, and extension services through affirmative action are essential steps. Gender‐transformative strategies must be woven into all sectors to challenge discriminatory norms, enhance women's decision‐making power within households and cooperatives, and alleviate the burden of unpaid care, such as through childcare support.

To effectively address the issue of declining dietary diversity, it is crucial to implement targeted nutritional security measures. Key strategies involve nutrition education initiatives that advocate for affordable and diverse diets using locally sourced foods, along with support for local production and marketing of nutritious foods, such as horticultural products and pulses. Furthermore, the potential introduction of targeted subsidies or vouchers for nutrient‐rich foods should be considered, especially for vulnerable households with young children, pregnant women, and lactating women.

## 6 Strengths and Limitations of the Study

This study employed rigorous and alternative econometric models to estimate the impact of food price changes on household food and nutrition security using a robust set of well‐established food and nutrition security indicators. A key strength of the analysis was the application of an extended version of the Oaxaca–Blinder decomposition technique adapted for panel data, which enabled a detailed examination of disparities in the impact of food price changes between female‐ and male‐headed households.

However, this study was limited by the unavailability of disaggregated food group price data at the regional level. Consequently, it was not possible to assess the differential effects of specific food group price changes (e.g., cereals, pulses, vegetables) on household food and nutrition security outcomes. Future research would benefit from incorporating food group–level price data to more precisely identify which types of food price fluctuations had the greatest impact on household food and nutrition security.

## Author Contributions

**Abule Mehare:** funding acquisition, project administration, supervision, data analysis, interpretation, and presentation of the manuscript on workshops. **Lamessa T. Abdisa:** conception, study design, critical review, revising, and editing of the previous version of the manuscript. **Shemelis Kebede Hundie:** data curation, formal analysis, methodology, writing – original draft, software, and review and editing.

## Ethics Statement

The authors have nothing to report.

## Consent

The authors have nothing to report.

## Conflicts of Interest

The authors declare no conflicts of interest.

## Data Availability

The data used in this study is the publicly available 2018/2019 and 2021/2022 Ethiopia Socioeconomic Survey (ESS) 2018/2019(ESS4) and is also available on reasonable request to the corresponding author.
